# Life power and art safeguard

**DOI:** 10.3389/fmicb.2014.00120

**Published:** 2014-03-26

**Authors:** Oana A. Cuzman

**Affiliations:** Institute for the Conservation and Valorization of Cultural Heritage-National Research CouncilFirenze, Italy

**Keywords:** biotreatment, art safeguard, bioremediation, biocleaning, bioconsolidation

When a works of art or a monument is completed, it actually starts a new life, a new life influenced by the overall context where this art piece will be placed and/or exhibited. It is like a newborn that starts its life adventure. The living context is not always favorable for the new entity, and different risks may occur, depending on the deterioration agents and the duration of the exposure.

Weathering, pollution, and biological colonization may cause serious problems to the exposed artistic entities. Could these deterioration problems be overcome by using sustainable and natural solutions, such as the help of living/dead microorganisms or only of their metabolic products?

The answer seems to be positive and quite promising, taking into account the potent features of the microorganisms, such as the minimum requirements for living, the high velocity of reproduction, the great number of the offspring and their great resistance and adaptability to adverse environmental factors. In fact, bioremediation of different degradation problems of works of art was already successfully proven (Ranalli et al., 2000, 2005; Joseph et al., 2012; Perito et al., 2013) with cleaning and consolidation treatments. One important fact must be considered when these treatments are used in outdoor conditions. This is related with the possible consequence of the biotreatment on the ecological equilibrium present already on the surfaces to be treated.

The naturally occurring microorganisms are always living in a dynamic equilibrium with the surrounding environment, forming communities of usually more than one species, so the new species used for the biotreatment could integrate or disrupt this balance. Even if dead microorganisms are used, they will surely become part of the ecosystem energy flow.

A biotreatment with living microorganisms at a big scale in an outdoor environment is definitely more difficult to control and understand its own behavior in time, being linked with the ecological factors. However, the using of isolated metabolic products rather than living or dead organisms are more appropriate, as they could be better controlled.

The thoroughly superpower of the microorganisms, if harnessed, could improve the safeguarding of works of art. To do this, it is essential to clearly individuate the cases where it could be applied, to have deep knowledge of the biotreatment trend and mechanisms, and of course, to ensure the reproducibility in similar conditions.
